# Seeking Inspiration: Examining the Validity and Reliability of a New Smartphone Respiratory Therapy Exergame App

**DOI:** 10.3390/s21196472

**Published:** 2021-09-28

**Authors:** Clarence Baxter, Julie-Anne Carroll, Brendan Keogh, Corneel Vandelanotte

**Affiliations:** 1 School of Public Health and Social Work, Faculty of Health, Queensland University of Technology, Kelvin Grove 4059, Australia; jm.carroll@qut.edu.au; 2 School of Public Health and Social Work, Institute of Health and Biomedical Innovation, Queensland University of Technology, Kelvin Grove 4059, Australia; 3 Digital Media Research Centre, Creative Industries, Education, and Social Justice Faculty, Queensland University of Technology, Kelvin Grove 4059, Australia; brendan.keogh@qut.edu.au; 4 Physical Activity Research Group, Appleton Institute, Central Queensland University, Rockhampton 4702, Australia; c.vandelanotte@cqu.edu.au

**Keywords:** telemedicine, mHealth, respiratory, smartphone, cellphone, digital simulation, microphone, gamification, serious game, exergame

## Abstract

Background: Clinically valid and reliable simulated inspiratory sounds were required for the development and evaluation of a new therapeutic respiratory exergame application (i.e., QUT Inspire). This smartphone application virtualises incentive spirometry, a longstanding respiratory therapy technique. Objectives: Inspiratory flows were simulated using a 3 litre calibration syringe and validated using clinical reference devices. Syringe flow nozzles of decreasing diameter were applied to model the influence of mouth shape on audible sound levels generated. Methods: A library of calibrated audio inspiratory sounds was created to determine the reliability and range of inspiratory sound detection at increasing distances separating the sound source and smartphones running the app. Results: Simulated inspiratory sounds were reliably detected by the new application at higher air inflows (high, medium), using smaller mouth diameters (<25 mm) and where smartphones were held proximal (≤5 cm) to the mouth (or at distances up to 50 cm for higher airflows). Performance was comparable for popular smartphone types and using different phone orientations (i.e., held horizontally, at 45° or 90°). Conclusions: These observations inform future application refinements, including prompts to reduce mouth diameter, increase inspiratory flow and maintain proximity to the phone to optimise sound detection. This library of calibrated inspiratory sounds offers reproducible non-human reference data suitable for development, evaluation and regression testing of a therapeutic respiratory exergame application for smartphones.

## 1. Introduction

At rest, normal human breaths are barely perceptible. Vigorous respiration produces audible sound at the mouth by means of turbulence created at higher air flow rates and greater air pressure changes [1,2]. Designed primarily for telephony and the capture of spoken voice sounds, built-in microelectromechanical (i.e., MEMS) microphones in contemporary smartphones can be repurposed to sense changes in air pressure arising from breath sounds. Diagnostic and therapeutic mHealth (i.e., mobile health) applications detect breath sounds and sound components for a variety of innovative health improvement purposes including respiratory rate monitoring in children, cough diagnostics, sleep apnoea detection, asthma inhaler technique training and respiratory function testing. [3,4,5,6,7,8,9].

Some mHealth apps infer respiratory flow rates by means of calibrated vortex whistles to produce sound of a known pitch for microphone detection, or by constraining and controlling mouth shape as a potential variable influencing the respiratory sounds generated; others may employ external microphones to improve sound detection [10,11]. Add-on components such as external microphones, whistles or spacers may add cost and complexity to mHealth apps or limit what phone types or models may be used, potentially limiting accessibility to patients, particularly in resource-poor or impoverished settings [12]. Built-in microphones are available in all smartphones and offer a single, easy to use encapsulated means for sound detection.

Serious gaming techniques (i.e., gaming for purposes other than entertainment) have been applied to mHealth application design in contexts such as disease detection and treatment, monitoring, health education and rehabilitation [13]. Gamification of mHealth apps offers an “attention shift” for users experiencing unpleasant or negative sensations such as breathlessness or fatigue [14]. Patients may be distracted from the repetitive nature of therapeutic exercise by engaging with engrossing and motivational game play [15].

This study concerns development and use of methodology for evaluating the validity and reliability of a new respiratory therapy exergame application for smartphones (i.e., QUT Inspire or Queensland University of Technology Inspire), using clinically valid and reproducible calibrated (non-human) simulated inspirations. This new smartphone application virtualises incentive spirometry (i.e., ISy), a longstanding respiratory therapy technique for mucus clearance from airways commonly employed in clinical contexts such as post-operative convalescence and in the management of certain chronic respiratory conditions such as chronic obstructive pulmonary disease (i.e., COPD) and cystic fibrosis [16].

**Figure 1:**
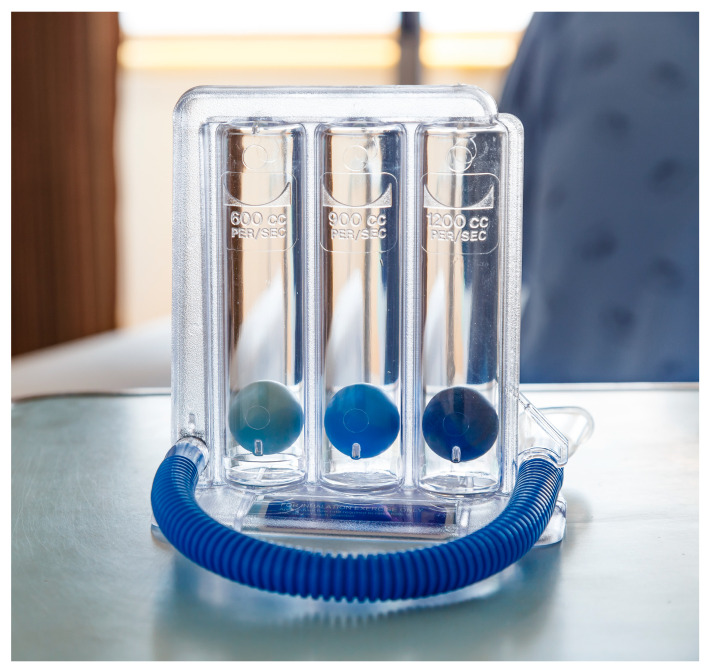
A Triflo II clinical incentive spirometer (image: iStock by Getty Images).

**Figure 2:**
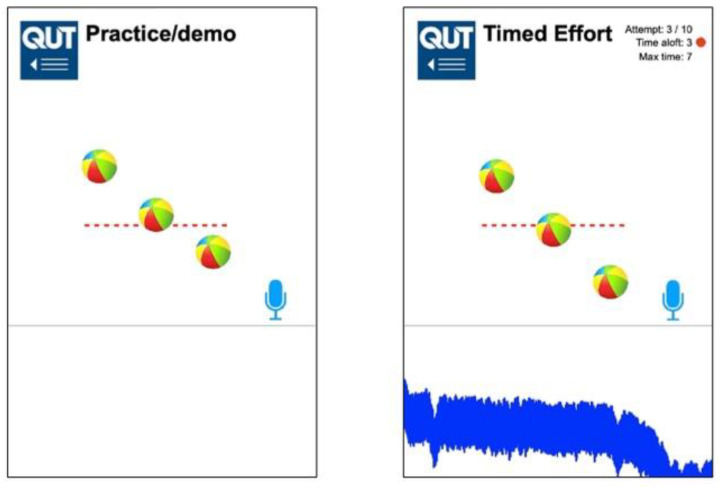
QUT Inspire incentive spirometer application—practice and timed modes.

From its inception, incentive spirometry was designed as a motivational affordance to encourage mucus clearance from the airways using repeated slow gradual maximal inspirations (Figure 1) [16]. An emergent role for ISy therapy has recently been proposed in pulmonary rehabilitation for convalescing SARS-CoV-2 (i.e., COVID-19) patients. [17]. In contrast to existing clinical incentive spirometer devices that levitate spheres or pistons in response to maximal inspiration, the new QUT Inspire smartphone application is an exertion-type exergame for the detection of inspiratory sound generated at the mouth using the smartphone microphone (without the need for any mouthpieces or other add-on components) [18]. The application displays an animated graphic on the screen to provide a responsive, visual incentive to persist with gradual, sustained, repeated inspirations (Figure 2). QUT Inspire is an exemplar of an exergame where a game is built into the interface, with a primary goal of keeping animated balls aloft by means of detecting inspiratory sound, and a secondary goal of encouraging sustained gradual maximal inhalation for mucus dislodgement from the airways and expulsion by coughing [19]. It has been argued that a safe and effective exergame must support primary and secondary goals defined for a therapeutic exercise to be safe and effective for patients [19].

Widespread availability of smartphone technology affords opportunities to leverage sensors built into these ubiquitous devices as a low cost, accessible means for improving health. However, risks to health exist where sensors in “badly behaving” apps may malfunction or misread physiological parameters, or where variations in application performance may be introduced by unintended or unforeseen user interactions with their phones while using a given app, or with updates to phone models, sensors or software that may affect application functionality [20]. This study aims to develop a library of reproducible, calibrated non-human simulated inspiratory audio samples representing a clinically valid range of inspiratory flow rates and mouth diameters, suitable for application development, testing and re-testing. The resultant audio library is applied to evaluation of the reliability and range of sound detection of the new QUT Inspire respiratory therapy exergame app.

## 2. Inspiratory Air Flow and Sound Generation

Mouth-throat modelling indicates that air flow near the mouth becomes turbulent at flow rates ranging from 15–45 L/min (i.e., approx. 250–750 cc/s) [21]. Sound levels greater than approximately 50 dBA become audible at the mouth as the inspiratory flow rate approaches 60 L/min (i.e., approx. 1000 cc/s) [22]. Inspiratory sounds are generally louder than those arising from exhalation [23]. Turbulent (and thus more audible) air flow requires a sustained air pressure difference between the outer atmosphere and mouth, proportional to the square of the flow rate [24,25]. Air flow resistance at the mouth contributes to sustaining this audible turbulence by constraining the diameter available for air exchange and increasing air pressure across the lumen of the mouth, with the resultant sound amplitude proportional to the square of the air flow at the mouth [26,27]. This is best demonstrated by pursing one’s lips (e.g., as in preparing to whistle) and noting an increase in audible mouth noise with boisterous inspiration. Respiratory sound amplitude diminishes as the distance between the sound source (i.e., mouth) and monitoring device is increased [27].

### 2.1. Sound Detection and Modern Smartphones

Only a small number of smartphone apps using built-in phone microphones meet international standards for sound level measurement, with fewer standards-compliant sound metering apps offered for Android smartphones compared with Apple devices [28]. This may be attributable to application development challenges arising from potential disparities in microphone architecture between multiple manufacturers of Android phones compared to a reported relative homogeneity of components in Apple devices [29,30]. The age of a given phone microphone may also influence monitored sound levels [29]. Sound detection in modern smartphones now encompasses voice-activated command features such as Siri which may implement sound-level filtering or attenuation to support voice detection when the phone is held proximal to, or at a distance from the mouth [31,32]. Potential variations in microphone performance between phone brands and between existing and emergent models highlight the need for analysis and benchmarking of sound detection, particularly where this is implemented in mHealth apps.

### 2.2. Limited Respiratory Application Availability for Improving Health

Several broad categories of smartphone respiratory monitoring apps have been identified including breathing pattern detection, cough sound analysis, sleep and snoring analysis, spirometry and lung function assessment [33,34]. Surveys of popular application stores (e.g., Apple App Store, Google Play Store) and curated mHealth application libraries (e.g., AppScript, MyHealthApps) yield a relative paucity of sensor-based respiratory mHealth apps translated from the research lab to offerings made available for prescription by clinicians or self-prescription by smartphone users [35,36]. Nevertheless, respiratory therapy apps may have application in both short- and long-term patient engagement, ranging from short hospital stays to protracted outpatient use for chronic conditions; treatment outcomes that previously required dedicated, and sometimes costly equipment may be realised by use of widely available smartphone technology [37,38]. Quality measures such as MARS (i.e., Mobile Application Rating Scale) for evaluating mHealth apps are gaining more widespread acceptance, particularly when vetting apps for inclusion in curated application libraries [39]. Technical validation of apps and clinical studies regarding application performance are requisite steps in building an evidence base supporting use of a given app, and in some countries such validation is a requisite for satisfying requirements for medical device registration [40]. Wider adoption of such mHealth apps may be contingent on rigorous evaluation and building an evidence base which demonstrates safety and effectiveness for an app, potentially contributing to the paucity of such apps offered by popular application stores [36,41]. 

### 2.3. Air Flows for Testing Respiratory Apps

It would be both unfeasible and unethical to expect human subjects to produce the considerably large number of reproducible breaths necessary to assert that new and emergent respiratory apps are both reliable (i.e., reproducible) and clinically valid when compared with reference devices [42]. Non-human generation of airflows for respiratory application testing has previously drawn on techniques used in the calibration of equipment in clinical respiratory laboratories settings [4,43]. Decompression calibrator devices and flow delivery pumps (for ventilator calibration) are specialist devices used in clinical respiratory laboratory settings to discharge air at pre-determined flow rates [44]. However, calibration syringes deliver known volumes of air by manual actuation of the syringe plunger to simulate inspiration or expiration. Syringes for generating air flows are relatively inexpensive compared with specialised devices, widely accessible and are a mainstay in clinical respiratory function testing settings for evaluating performance of respiratory function testing equipment (e.g., spirometers, flow meters, plethysmographs) [45,46].

Computerised respiratory sound analysis (i.e., CORSA) provides a foundation catalogue of abnormal respiratory sounds using standardised sound samples in apps for diagnosis or therapy [23,33]. Playback of sound recordings of inspiratory or expiratory air flows captured at known flow rates offers a reproducible means for assessing application performance, including the range (i.e., distance separating the phone from the sound source) and reliability of sound detection afforded by a given application [9,47]. Vortex whistle and other add-on devices have been employed in some apps to constrain mouth shape and translate airflow into sound of a known pitch, facilitating quantitating of air flows in peak flow measurement, metered dose inhaler medication training for asthma and in assessment of lung function [11,48,49]. A paucity of literature exists regarding the contribution of reduced mouth diameter to sound production during simulated inspiration, suitable for detection by smartphone microphones.

## 3. Incentive Spirometry and Respiratory Therapy

Incentive spirometry is a respiratory exercise intended to encourage repeated gradual maximal “purposeful” inspirations which result in intrapleural air pressure changes to precipitate mucus dislodgement and expulsion by coughing; the objective of this therapy is prevention of morbidity and mortality arising from respiratory infections and pneumonia [10,50]. ISy is employed in post-surgical convalescence (where anaesthesia or protracted periods of bed rest may result in mucus accumulation in the airways) and in management of some chronic disease conditions where mucus overproduction may occlude airways or increase the risk of respiratory infections such as COPD or cystic fibrosis [51]. In the presence of COPD, low inspiratory flow rates may also be a predictor for hospital re-admission following initial hospital admission for COPD [52,53]. Low inspiratory flow rates have been identified as a possible biomarker for COPD, offering a potentially inexpensive and accessible means for COPD screening [52,54]. 

ISy therapy has also recently been proposed for post-COVID-19 respiratory therapy and convalescence [17]. In the modern incarnation of this device, spheres or a piston embedded in a clear injection-moulded plastic chassis are attached to a breathing tube (Figure 1) [52]. The patient gradually and maximally inhales via the tube, creating a vacuum in the device chamber and levitating the spheres or piston. Flow-based and volume-based ISy devices are available for respiratory therapy, using either flow-rate calibrated levitation of a sequence of spheres or a graduated scale to report deflection of a piston and the volume of air inspired respectively [55].

### 3.1. QUT Inspire—A Virtual Incentive Spirometer

QUT Inspire is a virtualised incentive spirometer application for smartphones. It is a HTML5 web application developed using Construct 3 (scirra.com, Scirra Ltd., London, UK, accessed 17 September 2021) and designed for cross-platform compatibility with a range of popular smartphones using Apple IOS and Google Android operating systems, executed using the smartphone web browser.

The new application (Figure 2) detects inspiratory breath sound above a pre-determined threshold, using the built-in MEMS smartphone microphone as an uncalibrated pressure sensor. The lower part of the application screen displays sound frequency sampling as an indicator to the user that sound detection is active. The sound of inspiration triggers a three-ball animation (akin to the clinical flow-based ISy device depicted in Figure 1) in response to sustained inhalation effort. This animation is maintained while sound levels are sustained above a pre-determined threshold (i.e, 50 dBA). The height (i.e., elevation) of the spheres is a pre-set animation sequence of fixed magnitude; this does not reflect the actual flow rate values detected but instead reports to the user that sustained inspiratory sound is maintained and detected by the phone microphone. The sound level threshold can be altered using a slider control to minimise spurious triggering due to background noise.

Inspiratory air inflows were mechanically simulated using a 3 L calibration syringe (Welch Allyn Model: 7034803). The rate of withdrawal of the syringe plunger was timed using a counting sequence spoken out loud (Table 1) to generate a range of simulated inspiratory flow rates (high, medium and low).

### 3.2. Inspiratory Flow Rate (IFr)—Clinical Incentive Spirometer

To assess the clinical validity of the range of air inflows generated by this timed counting sequence, simulated inspiratory flows were applied to a clinical incentive spirometer device (Triflo II incentive spirometer Model: 8884717301), coupled to the syringe via a 3 m length of 25 mm internal diameter PVC hose. This hose length was selected to minimise any spurious audio artefacts arising from mechanical syringe actuation in the subsequent audio sampling of simulated inspirations. 

A 10 mm flow nozzle coupling connected the distal end of the hose to the Triflo II device, as the device offers a fixed 10 mm hose connection. For each flow rate, syringe-generated simulated inspirations (n = 15) were applied to the Triflo II device. Levitation of spheres in the device in response to syringe actuations was noted and compared with an inspiratory flow rate (i.e., IFr) scale embedded in the device chassis, indicating clinically significant air inflows (i.e., 600, 900 and 1200 cc/s).

### 3.3. Peak Inspiratory Flow Rate (PIFr)—Pneumotachograph

Reliability of mechanically simulated inspirations was assessed by attaching the calibration syringe to a Fleisch Type 2 pneumotachograph (Vitalograph Micro Model: 6300) and measuring peak inspiratory flow rate (i.e., PIFr). To model the effect of reduction in mouth diameter on the simulated inspiratory flow rates generated, flow reduction couplings of decreasing diameter (25, 20, 15 and 10 mm) were attached to the distal end of the PVC hose attached to the syringe (n = 15 syringe actuations at each flow rate and mouth diameter combination). These flow reduction couplings were hard neoprene washers, with a central hole of known diameter.

### 3.4. Sound Level of Syringe Inflows (dBA)—Digital Sound Meter

Sound levels generated by the mechanically simulated inspirations were measured with a digital sound meter (i.e., DSM) (Digitech Model: QM1591). Flow nozzles were clamped to a 1.5 m tall stand separated from another stand holding the DSM. Sound level measurements were repeated at increasing distances separating the flow nozzle and digital sound meter (1, 2, 5, 10, 20, 50 cm).

### 3.5. Acoustic Sampling for Smartphone Testing

To eliminate variability in flow rates generated, the sound produced by mechanically simulated inspirations was sampled with a digital sound recorder (i.e., DSR) (Sony Model:ICDPX470). Recordings were performed at a distance of 1 cm separation between the flow nozzle and the DSR to prevent spurious vibration of the recorder. The flow nozzle and sound recorder were clamped to individual stands at a height of 1.5 m above floor level. Audio frequency spectra for these digital sound samples were inspected (https://academo.org/demos/spectrum-analyzer/ accessed on 17 September 2021). A single audio sample was selected as representative for each flow rate and mouth diameter combination for application testing. For each sound sample, an audio loop of 15 iterations of a single audio sample inspiration was created to investigate the reliability of inspiratory sound detection. Five seconds of blank sound was interspersed in the audio loop between each simulated inspiration.

### 3.6. QUT Inspire Smartphone Application Testing

Audio sample loops were played back using a MacBook Pro computer via an external speaker (UE Roll Model:991-000105) for detection with the QUT Inspire application at increasing distances (1, 2, 5, 10, 20, 50 cm). Separating the speaker and smartphone. Sound level output from the speaker was checked and set to the same volume as that of the source sound generated by the syringe using the digital sound meter. Phones were oriented flat (horizontally) with the phone microphone pointed towards the speaker. Two contemporary smartphones were selected for evaluation of the new QUT Inspire app, an Apple iPhone XR (IOS 13.5.1) and Samsung Galaxy (Android 9.0 Pie). The smartphones were less than twelve months old. A separate test suite was conducted to assess the influence of phone orientation on detected simulated inspiratory sound, Both Apple and Android phones were oriented flat (horizontally) at 45° and 90° to the audio source respectively, to compare the range of detection of inspiratory sound by the QUT Inspire application at distances ranging from 1 to 50 cm separation between phone and audio source.

### 3.7. Statistical Analysis

IBM SPSS Statistics (version 26.0.0.1) was used to collate statistics and generate plots. Error bars on plots indicate 95% confidence intervals. Multiple linear regression was performed to model the influence of distance, flow rate and mouth diameter of mechanical syringe-generated inspirations on observed variability in PIFr and sound levels. Natural log transformations of variables (e.g., PIFr and sound level) were performed where exponential decay was observed over the distances investigated [56].

## 4. Results

### 4.1. Syringe-Generated Inspirations by Flow Rate—Triflo II Clinical ISy Device

**Figure 3:**
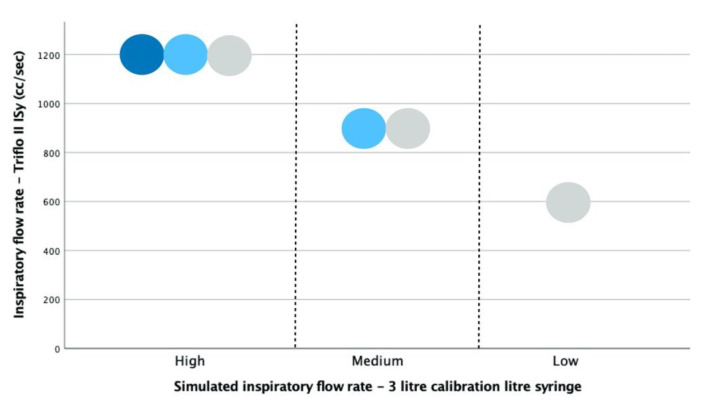
Triflo II ISy device—sphere(s) deflected with increasing inspiratory flow.

When mechanically simulated inspiratory flows were applied to the Triflo II ISy device at decreasing flow rates, inspiratory flow rates of 1200, 900 and 600 cc/s were reliably reported by this device (n = 15 of 15 simulated inspirations at each flow rate) (Figure 3). These syringe-simulated inspiratory flow values were within the range of clinically valid inspiratory flow rates the Triflo II clinical incentive spirometer device is designed to monitor. 

The Triflo II device offers a fixed 10 mm aperture for coupling to a breathing hose; only the 10 mm flow restriction nozzle was applied to the calibration syringe for the generation of simulated inspirations.

### 4.2. Peak Inspiratory Flow (PIFr) of Simulated Inspirations by Flow Rate and Mouth Diameter

**Figure 4:**
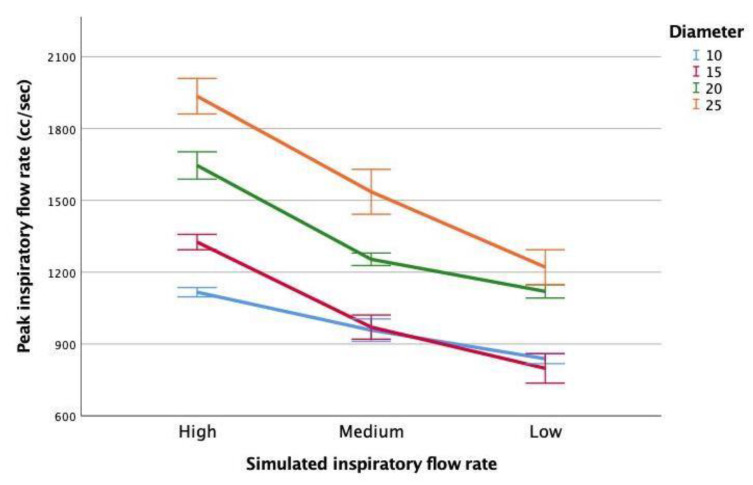
PIFr measured by pneumotachometer for syringe generated. Inspiratory flows by flow rate and mouth diameter.

The highest PIFr values recorded by a Fleisch Type II Pneumotachograph were observed for high simulated inspiratory flow rates and larger mouth diameters (e.g., 2000 cc/s at 25 mm mouth diameter) (Figure 4). Considering the fixed 10 mm aperture in the Triflo II Isy device, PIFr values measured by the pneumotachometer for the 10 mm mouth diameter yielded mean PIFr values of 1100, 900 and 800 cc/s for high, medium and low simulated inspiratory air flows respectively (n = 15 iterations at each flow rate and mouth diameter combination). These results are comparable to the clinically valid range of inspiratory flow rates displayed on the calibrated scale in the Triflo II Isy device (Figure 3).

Low simulated inspiratory flows measured as 600 cc/s by the Triflo II device, compared with 800 cc/s reported by the pneumotachometer. At medium and high simulated inspiratory flow rates, the simulated inspirations produced by timed withdrawal of a calibrated 3 L syringe plunger generated PIFr comparable to the clinically significant range by the calibration scale reported by Triflo II device. As the mouth diameter was increased beyond 10 mm, the PIFr also increased, with the highest PIFr values observed for the largest mouth diameter (25 mm).

The association of flow rate and mouth diameter with the observed peak inspiratory flow values was found to be significant (F_2,177_ = 553.381, *p* < 0.01), explaining 86.1% of the observed variability in peak inspiratory flow rate (adjusted R^2^ = 0.861). Both flow rate (ß_flow rate_ = −0.623, *p* < 0.01) and mouth diameter (ß_mouth diameter_ = 0.689, *p* < 0.01) contributed to the variability observed in this model. See Supplementary Materials file for the regression model. The highest simulated peak inspiratory flow rates were produced using higher mechanical syringe-simulated inspiratory flow rates (e.g., high) and application of larger mouth diameters (e.g., up to 25 mm).

### 4.3. Sound Levels of Syringe-Simulated Inspirations by Distance, Flow Rate and Mouth Diameter

**Figure 5:**
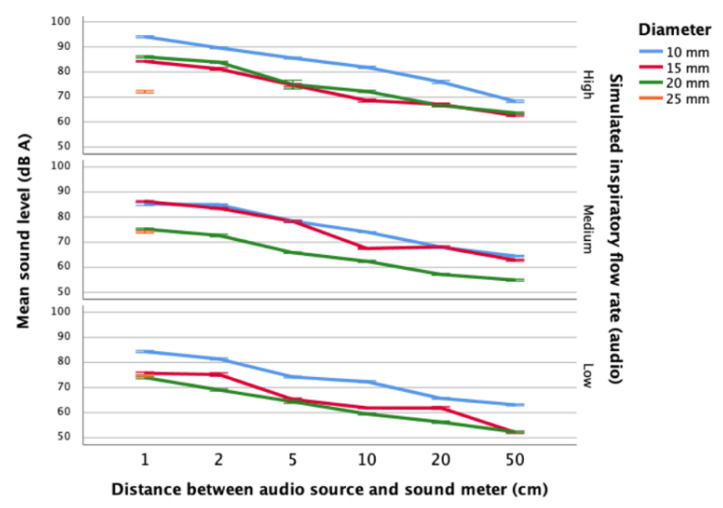
Sound levels of syringe-simulated inspirations by distance, flow rate and mouth diameter.

The sound generated by syringe-generated inspirations was measured using a digital sound meter for each flow rate and mouth diameter combination (Figure 5). Sound levels were monitored at increasing distances (separation) between syringe nozzle (simulated mouth) and DSM, akin to holding a phone further away from the mouth. In contrast to PIFr measurements where the largest mouth diameter generated the greatest flow rate, the highest sound levels (95 dBA) were measured where mouth diameter was small (i.e., 10 mm) (Figure 5). This may reflect turbulent, audible airflow where the lumen of the simulated mouth (nozzle) is small. Higher flow rates resulted in louder inspiratory sound. Sound levels detected by the DSM decreased as the distance separating the flow nozzle and the DSM was increased. The lowest sound levels (75 dBA) were observed where the largest mouth diameter (i.e., 25 mm) was used; no detectable sound was produced for this large mouth diameter at distances greater than 1 cm from the audio source.

The association of distance, flow rate and mouth diameter with sound levels arising from simulating inspirations using the calibration syringe was significant (F_3,851_ = 835.231, *p* < 0.01), accounting for 74.6% of the observed variability in sound levels (adjusted R^2^ = −0.746). See Supplementary Materials file for the regression model. 

Distance (ß_distance_ = −0.706, *p* < 0.00) explained more than twice the variability in sound levels compared with either flow rate (ß_flow rate_ = −0.374, *p* < 0.01) or mouth diameter (ß_mouth diameter_ = −0.398, *p* < 0.01). The highest peak inspiratory flow rates were measured when large mouth diameters (e.g., up to 25 mm) were applied using the pneumotachograph (Figure 4). Conversely, the highest sound levels arising from syringe-simulated inspirations were detected where the smallest mouth diameters (e.g., 10 mm) were used (Figure 5). Minimising the distance from the simulated “mouth” nozzle contributed more to producing higher and more audible inspiratory sound, with high syringe-simulated inspiratory flow rates and small mouth diameters also contributing to louder inspiratory sound for detection.

### 4.4. Sounds Level of Audio Samples of Syringe-Simulated Inspirations

**Figure 6:**
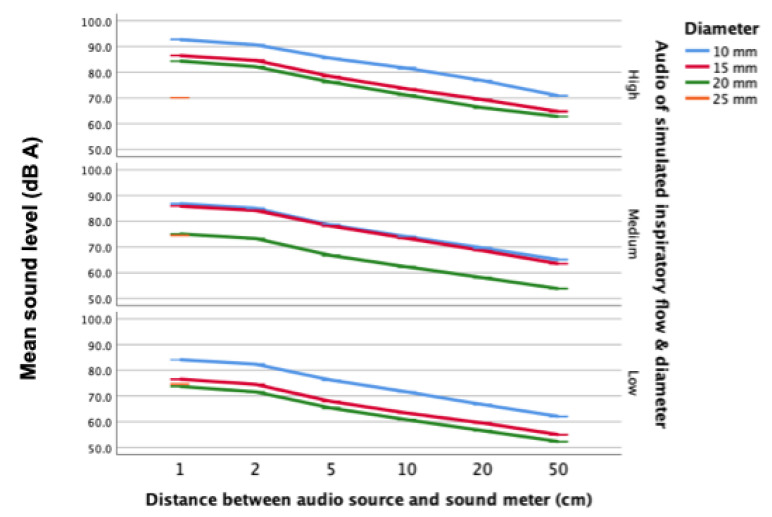
Sound levels of audio samples by distance, flow rate & mouth diameter.

Measured using a digital sound meter, sound levels for audio recordings of syringe-generated inspiratory sound were comparable with sound levels measured during syringe-simulated inspirations. (Figure 6).

The highest sound levels were recorded for high flow rate and small mouth diameter combinations (93 dB). Sound levels decreased as the distance between the audio source and digital sound meter increased. At each distance sampled (1, 2, 5, 10, 20 and 50 cm), higher airflows produced greater sound levels.

### 4.5. Audio Frequency Spectra for Simulated Inspirations

**Figure 7:**
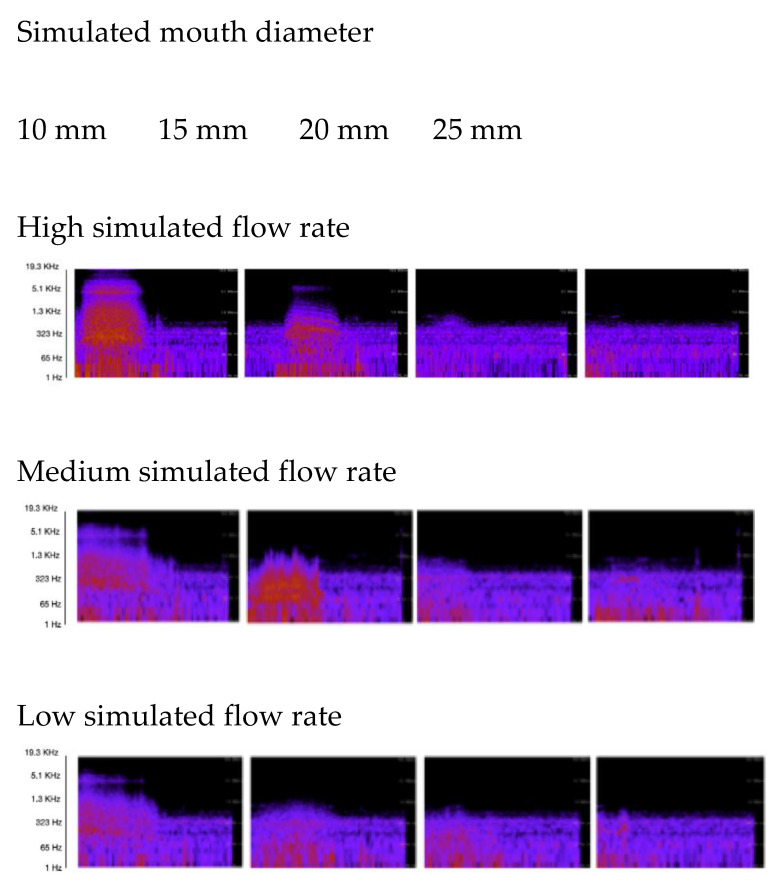
Audio frequency spectra for audio samples of simulated inspirations.

Audio spectra were generated for audio samples of each mechanically simulated inspiratory flow rate and mouth diameter combination, at a 1 cm distance from the audio source (Figure 7). Spectral peaks were greatest where mouth diameter was small (i.e., 10 mm) and the simulated inspiratory flow rate was high. Sound levels and resultant spectral peaks dissipated as flow rate was reduced, and as mouth diameter was increased. 

Considering the largest simulated mouth diameters (i.e., 20 and 25 mm), a clearly delineated spectral peak (or perceptible sound) was difficult to identify. A representative sample of each mechanically simulated flow rate and mouth diameter combination was selected for testing the new QUT Inspire app.

### 4.6. QUT Inspire Application Testing with Audio Samples

Table 2 summarises investigations regarding the range of inspiratory sound detection of audio samples by Android and Apple smartphones running the new QUT Inspire application according to the increasing distance between the audio source and the smartphone. Results indicate that high and medium flows using mouth diameters of less than 25 mm were reliably detected at distances of up to 50 cm from the audio source. Low inspiratory flows were detectable at distances up to 5 cm using 10 or 15 mm mouth diameters for inspiration and up to 50 cm where mouth diameter was 10 mm. 

Inspirations simulated with the largest mouth diameter (25 mm) were not detected at any of the distances examined. The range of detection of inspiratory sounds by the QUT Inspire application was unchanged when the phone orientation relative to the sound source was rotated from horizontal to 45° and 90°, respectively.

## 5. Discussion

The need for rigorous evaluation of mHealth apps has been mandated by the World Health Organisation and other stakeholders in light of the proliferation and popularity of health apps, and a paucity of evidence regarding application safety and efficacy. [20,39,40,41]. Widespread adoption of sensor-rich smartphones offers potential for generating and leveraging health data for health improvement purposes, notwithstanding the need for scrutiny to assert the validity and reliability of these emergent tools [34,36].

Complex sensor data can be simplified and presented to users in the form of exergames, using on-screen avatars or animations to respond to body movements (i.e., limb movements, respirations) in virtual or augmented environments designed to motivate engagement with a game and achieve some form of health improvement goal by engaging with game play [57]. In the context of exergaming, evidence is required to assert that both the primary and secondary goals for a given therapeutic exergame are achieved when using a new app, and that efficacy and safety are maintained when translating a conventional longstanding exercise technique or therapy to a virtualised implementation of that therapy [19]. In this study, a new smartphone respiratory therapy mHealth application for virtualising incentive spirometry (QUT Inspire) was investigated using non-human airflows generated with a calibration syringe to assess the reliability of inspiratory sound detection and validity compared with clinical reference measures.

This study contributes knowledge regarding the primary goal for this new application, namely reliable detection of inspiratory sound to trigger the display of an animation at a range of distances between phone and user, and with the application of different air inflow rates and simulated mouth diameters. Demonstration of the reliable triggering of the motivational animation at a range of distances contributes information regarding the safety of the new application. While the primary goal for this therapeutic exergame application is engaging and “distracting” the user to keep animated spheres aloft using the smartphone microphone to detect breath sounds, the secondary (and therapeutic) goal is the encouragement to persist with and to repeat gradual maximal inspiration to promote mucus clearance from the airways. The next step in these investigations are clinical studies to demonstrate the comparative efficacy of the new smartphone application compared with clinical devices in disease-free human subjects, and then in patients with disease.

Key insights arising from this research include highlighting the importance of sustaining a sufficiently high inspiratory flow rate and maintaining a small mouth diameter during inspiration to generate the largest, most audible sound levels. Further, the benefit of keeping the phone close to the mouth to maximise inspiratory sound detection was also demonstrated. As a precursor to in vivo trials of this new app, insights gained from these studies inform enhancements to the application interface design such as instructional on-screen prompts to coach users to maintain a small mouth diameter and minimise the distance between the user’s mouth and the phone microphone to optimise sound detection. Performance of the new application was comparable on examples of both Apple and Android smartphones, with no difference found in the range of simulated inspiratory sound detection when smartphone orientation was varied (i.e., held flat or horizontal, at 45 or at 90 degrees from the sound source).

Contemporary clinical incentive spirometers are robust mechanical devices that utilise a vacuum created by gradual maximal inspiration to levitate spheres (i.e., flow-based) or deflect a piston (i.e., volume-based) in a transparent plastic chassis, motivating compliance and persistence with repeated inspiratory efforts for therapeutic benefit by dislodging mucus from the airways for expulsion by coughing [16,53]. Debate exists as to the comparative clinical efficacy of these two types of ISy device; both device types are employed in clinical respiratory therapy [16,52]. Previous studies of incentive spirometry in healthy subjects suggest that higher inspiratory flow rates may be a more significant determinant of chest wall expansion than the type of incentive spirometry device [58].

In contrast to vacuum-triggered ISy devices such as the Triflo II, the new QUT Inspire virtual ISy application detects inspiratory sound using the built-in smartphone microphone displaying a levitating sphere animation on the screen (Figure 2), akin to the Triflo II flow-based ISy device (Figure 1). Turbulent airflows have been recorded at the mouth, with inspiratory flow becoming audible from 250 to 750 cc/s [21,22]. Acoustic recordings of breath sound components have been employed in the evaluation of smartphone mHealth apps previously, with respiratory sound components played back in contexts as diverse as cough sound analytics, snoring and sleep apnoea diagnosis; post-processing and complex analytics applied to sound samples yield diagnostic or therapeutic information without the need for accurate quantitation of respiratory flow rates per se [6,7,34,43].

Unlike classification of patterns of coughs or snoring, apps for performing respiratory function testing require the ability to quantitate flow rates, both for reporting clinically significant respiratory flows and for estimation of lung volumes (i.e., calculating flow over time). For example, a smartphone respiratory function testing application called Spirosmart was evaluated using playback of sound recordings of expirations captured from 52 subjects, comparing results with formal clinical respiratory function testing for each subject [9]. To improve measurement accuracy, users breathed through a plastic spacer mouthpiece device at the time of sound capture to constrain and standardise the diameter and shape of the mouth (for flow rate calculation) [9,48]. The investigators in this study reported successful sound detection by this application at distances up to arm’s length separating the phone from the mouth [47]. Respiratory rate estimation has also been performed using smartphone microphone recordings [41]. Breath sounds were recorded at tracheal and nasal sites using built-in and headset smartphone microphones [46]. Nasal sound monitoring was found to be superior to sound sampling at the trachea in this study with respiratory rates accurately estimated at distances of up to 30 cm separating the nose from the smartphone microphone [46]. A separate investigation of cough sounds demonstrated a reliable range of detection for expiratory cough sounds at distances up to 30 cm separation [27].

Add-on devices such as spacers, whistles and headset microphones may present barriers to wider adoption of mHealth apps due to factors such as cost, compatibility and availability [12]. For the purpose of detection of gradual maximal inspiration in the context of ISy therapy, the QUT Inspire application does not quantitate respiratory airflows. The new application detects inspiratory sound and sustains a display animation while sound persists. The range of sound detection for the new application was comparable to results of previous studies reporting breath sound detection up to an arm’s length separating user and phone for respiratory function testing, respiratory rate estimation or cough detection [9,27,47]. In this study, high and medium airflows using smaller mouth diameters were detected up to 50 cm from the sound source by the Android and Apple smartphones tested.

The simulation equipment and testing environment present several limitations to the applicability of these study results to use in human subjects. The calibration syringe used in these simulations has a capacity of 3 L; this may not be representative of reduced lung capacities encountered with some respiratory conditions. Smaller syringes are available for regression testing to mitigate this risk. The flow reduction nozzles used for modelling changes in mouth diameter were neoprene washers, each possessing a single central circular hole of known diameter. Characteristics of turbulent air inflows (and audible sound) generated using these symmetrical apertures may differ when more complex (i.e., non-circular) mouth shapes may be formed when humans reduce their mouth shape. A smartphone held in the hand may distort the sound detected by a built-in microphone (e.g., if the hand is cupped around the phone producing an echo or sound attenuation effect). Background noise such as that encountered in hospital wards or shared living environments may also affect the detection of inspiratory sound. The QUT Inspire application displays a sliding onscreen volume control for suppression of background noise. Testing this background noise suppression feature was outside the scope of this research. While both smartphones selected for this study (i.e., Apple and Android) were less than 12 months old at the time of testing, newer phone models are often released. It is envisaged that audio samples such as those produced for this research may be amenable for regression testing newer phones to assess performance in light of advances in phone hardware and software.

Smartphones contain additional sensors that may complement or enhance future iterations of respiratory therapy mHealth apps such as the new QUT Inspire app. Built-in proximity (or approach) sensors measure the distance between a phone and user to unlock or brighten the phone screen as the phone is moved closer to the head, a feature that may be used to alert the user if the phone is too far away from the mouth and optionally prompt the user to bring the phone closer to their head to improve sound detection [3]. Advanced camera sensor technology in modern smartphones (e.g., Apple Face ID) is capable of measuring mouth diameter [59]. This measurement could prompt the user to adopt a smaller mouth shape to maximise sound production. These ancillary sensors may contribute to addressing limitations to the range of detection and effect of mouth diameter identified in this research. Recent research in gait analysis using built-in accelerometer sensors in smartphones shows promise in estimating a subject’s lung capacity based on elements derived from their walking pattern [21]. These estimates could be used to customise respiratory effort required for inspirations or to display the normal range of respiratory values calculated for a given user (i.e., prediction of total lung capacity based on height, as inferred by gait) as part of the screen display of apps such as QUT Inspire.

## 6. Conclusion

A methodology for the acoustic sampling of mechanically simulated inspirations is presented here, with inspirations generated by a calibrated syringe at a range of clinically valid flow rates using a selection of mouth diameters of decreasing size (25–10 mm) to model conditions for audible inspiratory sound generation. Audio of these inspirations is used to evaluate the reliability and range of inspiratory sound detection for a new smartphone mHealth app that virtualises incentive spirometry. This methodology may also be amenable for evaluating other types of diagnostic or therapeutic respiratory mHealth apps. Results from this study inform new and emergent application design requirements such as the addition of screen prompts to remind users to maximise inspiratory flow rate, to reduce mouth diameter to maximise audible inspiratory sound production and to minimise the distance between the user and their phone to optimise inspiratory sound detection. Opportunities exist to incorporate other built-in phone sensors (e.g., camera, proximity and accelerometer sensors) as adjuncts to improving detection and display of inspiratory sound.

## Figures and Tables

**Table 1:** Counting sequence for syringe withdrawal.

Flow Rate	Counting Sequence
High	One, two, three
Medium	One, one hundred, two, one hundred
Low	One, one hundred thousand, two, one hundred thousand

**Table 2:** QUT Inspire application—range of inspiratory sound detection by flow rate and mouth diameter. When the following symbols are shown it means sound was detected using the corresponding simulated mouth diameter.

Distance Between Smartphone and Audio Source						
	1 cm	2 cm	5 cm	10 cm	20 cm	50 cm
High flow audio						
Apple iPhone XR	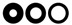	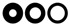	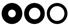	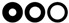	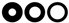	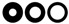
Samsung Galaxy S10	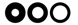	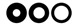	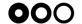	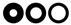	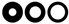	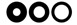
Medium flow audio						
Apple iPhone XR	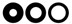	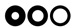	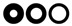	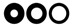	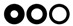	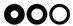
Samsung Galaxy S10	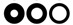	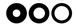	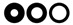	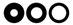	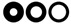	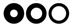
Low flow audio						
Apple iPhone XR	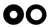	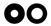	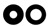	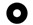	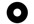	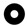
Samsung Galaxy S10	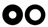	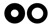	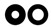	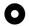	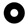	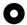

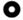
 = 10 mm; 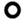 = 15 mm; 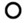 = 20 mm; 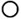 = 25 mm.

## Data Availability

The data presented in this study are available on request from the corresponding author. The data are not publicly available due to institutional policy.

## Supplementary Materials

Regression models—Model 1 (Section 4.2) and 2 (Section 4.3). https://www.mdpi.com/article/10.3390/s21196472/s1.
