# Supplementary material for: Seeking Inspiration: Examining the Validity and Reliability of a New Smartphone Respiratory Therapy Exergame App
Source: Sensors (Basel). 2021 Sep 28;21(19):6472. doi: 10.3390/s21196472 (PMC8513019; doi:10.3390/s21196472)
Supplement: Supplementary file 1 [file sensors-21-06472-s001.zip › sensors-1335978-supplementary.pdf]

Supplementary Material

SPSS Regression Model: Peak Inspiratory Flow – Pneumotachometer

Refer to section 4.2 of manuscript

Variables Entered/Removed[a]

Model	Variables Entered	Variables Removed	Method
1	FLOW_RATE, DIAMETER[b]	.	Enter

a. Dependent Variable: LN_PIF_PNEUMO

b. All requested variables entered.

Model Summary

					Change Statistics				
Model	R	R Square	Adjusted R Square	Std. Error of the Estimate	R Square Change	F Change	df1	df2	Sig. F Change
1	.929[a]	.862	.861	.10118	.862	553.381	2	177	<.001

a. Predictors: (Constant), FLOW_RATE, DIAMETER

ANOVA[a]

Model		Sum of Squares	df	Mean Square	F	Sig.
1	Regression	11.330	2	5.665	553.381	<.001[b]
	Residual	1.812	177	.010		
	Total	13.142	179			

a. Dependent Variable: LN_PIF_PNEUMO

b. Predictors: (Constant), FLOW_RATE, DIAMETER

Coefficients[a]

		Unstandardized Coefficients		Standardized Coefficients		
Model		B	Std. Error	Beta	t	Sig.
1	(Constant)	-.003	.031		-.084	.933
	DIAMETER	.033	.001	.689	24.676	<.001
	FLOW_RATE	-.206	.009	-.623	-22.313	<.001

a. Dependent Variable: LN_PIF_PNEUMO

Supplementary Material

SPSS Regression Model: Sound level - Syringe-simulated inspirations

Refer to section 4.3 of manuscript

Variables Entered/Removed[a]

Model	Variables Entered	Variables Removed	Method
1	FLOW_RATE, DIAMETER, DISTANCE[b]	.	Enter

a. Dependent Variable: VALUE

b. All requested variables entered.

Model Summary[b]

Model	R	R Square	Adjusted R Square	Std. Error of the Estimate	Durbin-Watson
1	.864[a]	.746	.746	4.9433	.096

a. Predictors: (Constant), FLOW_RATE, DIAMETER, DISTANCE

b. Dependent Variable: VALUE

ANOVA[a]

Model		Sum of Squares	df	Mean Square	F	Sig.
1	Regression	61230.793	3	20410.264	835.231	<.001[b]
	Residual	20795.618	851	24.437		
	Total	82026.410	854			

a. Dependent Variable: VALUE

b. Predictors: (Constant), FLOW_RATE, DIAMETER, DISTANCE

Coefficients[a]

Model		Unstandardized Coefficients		Standardized Coefficients			Correlations			Collinearity Statistics	
		B	Std. Error	Beta	t	Sig.	Zero-order	Partial	Part	Tolerance	VIF
1	(Constant)	99.892	.754		132.487	.000					
	DISTANCE	-.410	.010	-.706	-40.746	<.001	-.671	-.813	-.703	.992	1.008
	DIAMETER	-.854	.037	-.398	-22.941	<.001	-.335	-.618	-.396	.992	1.008
	FLOW_RATE	-4.484	.207	-.374	-21.656	<.001	-.374	-.596	-.374	1.000	1.000

a. Dependent Variable: VALUE

Collinearity Diagnostics[a]

Model	Dimension	Eigenvalue	Condition Index	Variance Proportions			
				(Constant)	DISTANCE	DIAMETER	FLOW_RATE
1	1	3.325	1.000	.00	.03	.01	.01
	2	.526	2.514	.00	.93	.01	.02
	3	.116	5.345	.02	.00	.23	.76
	4	.033	10.070	.98	.04	.75	.21

a. Dependent Variable: VALUE

Residuals Statistics[a]

	Minimum	Maximum	Mean	Std. Deviation	N
Predicted Value	48.834	86.455	71.936	8.4675	855
Residual	-12.0705	11.1273	.0000	4.9347	855
Std. Predicted Value	-2.728	1.715	.000	1.000	855
Std. Residual	-2.442	2.251	.000	.998	855

a. Dependent Variable: VALUE
